# Paired Associative Stimulation Targeting the Tibialis Anterior Muscle using either Mono or Biphasic Transcranial Magnetic Stimulation

**DOI:** 10.3389/fnhum.2017.00197

**Published:** 2017-04-20

**Authors:** Natalie Mrachacz-Kersting, Andrew J. T. Stevenson

**Affiliations:** Center for Sensory-Motor Interaction (SMI), Department of Health Science and Technology, Aalborg UniversityAalborg, Denmark

**Keywords:** human, paired associative stimulation, transcranial magnetic stimulation, biphasic current, plasticity, tibialis anterior

## Abstract

Paired associative stimulation (PAS) protocols induce plastic changes within the motor cortex. The objectives of this study were to investigate PAS effects targeting the tibialis anterior (TA) muscle using a biphasic transcranial magnetic stimulation (TMS) pulse form and, to determine whether a reduced intensity of this pulse would lead to significant changes as has been reported for hand muscles using a monophasic TMS pulse. Three interventions were investigated: (1) suprathreshold PA_bi_-PAS (*n* = 11); (2) suprathreshold PA_mono_-PAS (*n* = 11) where PAS was applied using a biphasic or monophasic pulse form at 120% resting motor threshold (RMT); (3) subthreshold PA_bi_-PAS (*n* = 10) where PAS was applied as for (1) at 95% active motor threshold (AMT). The peak-to-peak motor evoked potentials (MEPs) were quantified prior to, immediately following, and 30 min after the cessation of the intervention. TA MEP size increased significantly for all interventions immediately post (61% for suprathreshold PA_bi_-PAS, 83% for suprathreshold PA_mono_-PAS, 55% for subthreshold PA_bi_-PAS) and 30 min after the cessation of the intervention (123% for suprathreshold PA_bi_-PAS, 105% for suprathreshold PA_mono_-PAS, 80% for subthreshold PA_bi_-PAS. PAS using a biphasic pulse form at subthreshold intensities induces similar effects to conventional PAS.

## Introduction

Paired Associative Stimulation (PAS), introduced by Stefan et al. (2000), consists of the repetitive pairing of a peripheral electrical and a central magnetic stimulus at low frequency. Typically, the first stimulus is a single electrical pulse applied to the peripheral nerve innervating the target muscle, followed by a second stimulus applied using transcranial magnetic stimulation (TMS) over that area of the motor cortex that has direct corticospinal projections to the target muscle. Depending on the relative timing between these two stimuli, the direction of the synaptic change is either one of potentiation or depression (Wolters et al., 2003). PAS is based on studies of associative long term potentiation (LTP) and depression (LTD) in animal models (Bi and Poo, 1998) where the activation of presynaptic and postsynaptic neurons correlated in time, is artificially induced and the continued pairing of these two events leads to a strengthening of the synapse that outlasts the period of stimulation. Like LTP, PAS effects are dependent on the activation of NMDA-receptors and involvement of (L-type voltage-gated) Ca^2+^ channels (Stefan et al., 2002; Wolters et al., 2003). In this way, Stefan et al. (2002) were able to demonstrate that indeed many components of PAS resemble those of LTP. LTP is one mechanism for inducing synaptic plasticity thought to underlie processes of memory storage and learning (Letzkus et al., 2007).

Since these initial reports, PAS has been applied to numerous target muscles located in the hand (Ridding and Taylor, 2001; Ridding and Uy, 2003; Fratello et al., 2006; Kujirai et al., 2006; Quartarone et al., 2006; Ridding and Flavel, 2006; Rosenkranz and Rothwell, 2006; Roy et al., 2007), to those in the lower limb (Stinear and Hornby, 2005; Prior and Stinear, 2006; Mrachacz-Kersting et al., 2007; Roy et al., 2007; Kumpulainen et al., 2012, 2015; Mrachacz-Kersting, 2013) and in a variety of patient populations (Quartarone et al., 2003; Uy et al., 2003; Bagnato et al., 2006; Weise et al., 2006; Castle-Lacanal et al., 2007). However, different protocols make direct comparisons between these studies difficult. For example, while in hand muscles, PAS can have an effect in the relaxed muscle and with a standard interstimulus interval (ISI) between the peripheral and central stimulus across participants (Stefan et al., 2000). PAS applied to lower limb muscles such as the tibialis anterior (TA) requires either a pre-activated muscle or an individualized ISI to have a significant effect (Mrachacz-Kersting et al., 2007; Kumpulainen et al., 2012). In 2008, the first consensus article on motor cortex plasticity protocols was published (Ziemann et al., 2008) that highlighted the importance of investigating the effects following PAS in providing further information on mechanisms of memory formation and learning in the intact human. At this time only two studies with lower limb muscles as the target of PAS were cited (Mrachacz-Kersting et al., 2007; Roy et al., 2007). It is well known that the organization of neural pathways of lower limb muscles differs from that of the upper limb muscles. For example, motor evoked potentials (MEPs) in hand muscles are transiently inhibited by an afferent volley arriving at the sensory cortex following median nerve stimulation (Tokimura et al., 2000) while the afferent volley from the tibial nerve facilitates both the TA and Soleus MEPs (Roy and Gorassini, 2008). Furthermore, the I waves which are thought to be involved in the effects following PAS are predominantly I3 waves (Kujirai et al., 2006). These are readily elicited in upper limb muscles at TMS intensities around motor threshold when the current induced in the brain flows from anterior to posterior (Sakai et al., 1997). For lower limb muscles however it is not possible to preferentially recruit I3 waves (Di Lazzaro et al., 2001b).

The representation of the TA on the motor cortex is buried in the interhemispheric fissures, whereas that of hand muscles lie closer to the surface (Rothwell, 2003). A stronger magnetic field is required to induce an electric current to stimulate neurons in the cortical representation of TA. Evidence suggests that the stimulus efficacy is higher, both in terms of the threshold for excitation as well as response size, for a biphasic pulse waveform when TMS is applied (Maccabee et al., 1998). Further, a biphasic current will activate neurons within the cortex that are orientated in the AP as well as the PA direction and thus may not activate the same neural elements as a monophasic pulse form. It has also been argued that the biphasic pulse form may activate different sets of neurons with different thresholds (Kammer et al., 2001; Sommer et al., 2006), compared to the monophasic pulse form. It may thus be speculated that biphasic TMS might activate a more diverse set of cortical neurons than monophasic TMS. Indeed, Arai et al. (2005, 2007) suggested that during biphasic repetitive TMS (rTMS), several different populations of neurons are activated as compared to monophasic rTMS which activates only one population of neurons oriented in one direction. Whether this also leads to more effective alterations in excitability is not known.

There were two aims of this study: first, to establish whether PAS using either a suprathreshold monophasic or biphasic TMS stimulus will result in similar changes in the excitability of the cortical projections to the human TA muscle when applied at rest. Second, to determine whether it is possible to induce similar effects with PAS using a subthreshold biphasic TMS compared to a suprathreshold biphasic TMS pulse.

## Materials and Methods

### Participants

All participants in Experiment 1 and 2 provided written and informed consent in accordance with the Declaration of Helsinki to participate in this study. Approval was given by the Scientific Ethics Committee of Northern Jutland (Reference number: VN-20070015). All participants were classified as right side dominant according to the Edinburgh handedness inventory questionnaire (Oldfield, 1971) with a mean laterality quotient of 0.95 (range: 0.56–1). At the time of the study, all participants were free of any known physical or neurological disorders.

### Apparatus and Instrumentation

Surface electrodes (20 mm Blue Sensor Ag/AgCl, AMBU A/S, Denmark) were used to record the electromyographic (EMG) activity of TA and soleus (SOL) of the right leg for all aspects of the experiments. The electrodes were placed in accordance with recommendations of Cram and Kasman (1998). All data were sampled at a frequency of 4 kHz. Post-acquisition, the EMG signals were amplified and band pass filtered at 20 Hz–2 kHz offline.

### Stimulation

Depending on the experimental protocol, either a Magstim 200 or a MagstimRapid^2^ (Magstim Company, Dyfed, UK) with a focal figure of eight double-cone coil (110 mm diameter) was used to apply single pulses to elicit a MEP in the muscle of primary interest which was the right TA muscle. The direction of the current flow across the motor cortex was directed from posterior to anterior.

Stimulation of the right common peroneal nerve (CPN) was applied using a NoxiTest isolated peripheral stimulator (IES 230). Stimulating electrodes (32 mm, PALS® Platinum, Patented Conductive Neurostimulation Electrodes, Axelgaard Manufacturing Co., Ltd., Fallbrook, CA, USA) were placed on the skin overlying the deep branch of the right CPN (L4 and L5) with the cathode proximal. A suitable position for stimulation, defined as the site where a maximal M-wave was produced in the TA with no activity from the synergistic peroneal muscles and no activity from the antagonist SOL, was located. Palpation of SOL and peroneal muscles was performed during stimulation trials to ensure that this was occurring. This site corresponded to a point just anterior to the level of the caput fibulae. The pulse width was set to 1 ms and the intensity to 1× motor threshold. Motor threshold was defined as that intensity of stimulation where an M-wave first became visible in the EMG traces. Pilot studies revealed that these pulse settings produced the most consistent changes when combined with TMS (Mrachacz-Kersting et al., 2007).

### Somatosensory Evoked Potentials

One week prior to the main experiment, the cortical potentials evoked by the imposed stimulation of the right CPN were recorded with surface disc electrodes (E21-9 Disk Electrode—Standard 9 mm tin cup, Cephalon, DK) placed on the scalp, according to the International 10-20 system (Yamada, 2000). Somatosensory evoked potentials (SEPs) were recorded with a vertex electrode placed over CP_z_ and one placed over CP_2_ (band pass, 0.05–1000 Hz; sampling rate, 10 kHz, referenced to F_z_). A minimum of 1000 (maximum 3000) traces were recorded and ensemble averaged online. The characteristics of the pulse were the same as those used during the application of PAS (width of 1 ms, intensity of 1× motor threshold). The arrival of the evoked potential was measured as the time of occurrence of the first negative peak as has been done previously (Mrachacz-Kersting et al., 2007).

### Experimental Procedures

Participants were seated in a fixed chair (Hip 90°, Knee 130°) with their right foot resting on a moveable footplate. Initially, the stimulation intensity for the TMS was set at approximately 50% of maximum stimulator output (MSO) to find the optimal site for evoking a MEP in the right TA. The hot-spot was taken as the coordinate where the peak-to-peak amplitudes of the MEPs was greater in the target muscle than amplitudes of adjacent coordinates for a given stimulus intensity. For all participants, this site was approximately 2–3 cm anterior to the vertex and a stimulation applied to this area also evoked a response in the SOL. Once the hot-spot was identified, it was stored in Brainsight (Brainsight^TM^ version 1.5. Rouge Research Inc., Montreal, QC, Canada). This program was used throughout the experiment to ensure the coil position was maintained so that the stimulation was always applied over the same area of the motor cortex.

Subsequently, the resting motor threshold (RMT), defined according to the recommendations of the IFCN Committee (Chen et al., 2008) as the highest stimulus intensity that produced no more than 5 of 10 consecutive TA MEPs with an amplitude of ~50 μV while the muscle was at rest, was identified. Unless otherwise stated, all subsequent stimuli were delivered at 120% of RMT to ensure TA peak-to-peak MEP amplitude of approximately 0.5 mV. However, due to the location of the TA representation on the motor cortex, it was not possible to attain an MEP amplitude of 0.5 mV in all participants even when the intensity of the TMS pulse was increased. As a consequence, the amplitude value varied from 0.1 mV to 0.9 mV across all participants. For Experiment 2 (see below), the active motor threshold (AMT) was identified. This was defined as the highest stimulus intensity that produced no more than 5 out of 10 consecutive TA MEPs with an amplitude of ~200 μV while the muscle was contracting at 5% of its maximum voluntary contraction (MVC; Rossini et al., 1994; Chen et al., 2008). In all experiments, the participants were initially asked to perform a MVC of the TA to determine the maximum force which the participants was able to voluntarily exert at the plate. Participants were initially asked to contract their TA as hard as possible. Participants were instructed to pull their toes upwards as powerfully as possible on the word “go”, and to maintain this position until instructed to relax after 2–3 s. They were then allowed to relax for 1–3 min prior to the next trial. The best of a total of three collected trials was deemed the participants’ MVC. The root mean square (RMS) value of the rectified TA EMG for the MVC over a 1 s period was calculated. Subsequently, the participants were provided with visual feedback via a computer screen displaying a horizontal marking set at 5% MVC and a vertical bar displaying the participants’ current level of TA activation. Participants were asked to maintain the bar at the horizontal marking while AMT was identified.

### Paired Associative Stimulation (PAS) Protocol

**Figure 1:**
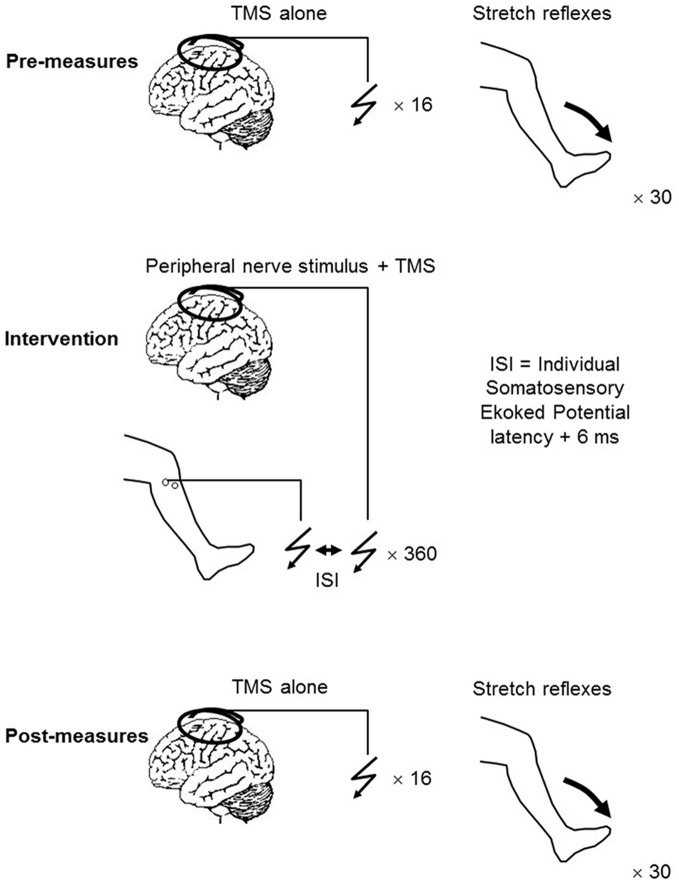
**Experimental protocol**.

PAS consisted of a single electrical stimulation of the CPN delivered at motor threshold, followed by a single TMS pulse delivered to the motor cortex. Depending on the experiment (i.e., the PAS protocol), the intensity of this was either 120% RMT or 95% AMT. A total of 360 pairs of stimuli were applied at a rate of 0.2 Hz (Figure 1).

### Experiment 1a: The Effect of Suprathreshold Monophasic Compared to Suprathreshold Biphasic TMS on PAS Induced Changes

To address the first aim, a total of 11 participants took part in Experiment 1 (9 males, 2 females, age 27 ± 5 years), which consisted of two interventions being applied to the participants as outlined in Figure 1. One week elapsed between each intervention. The TMS pulse used during the intervention was always set at 120% RMT and the current across the motor cortex directed from posterior to anterior (PA), however, the pulse type was either monophasic or biphasic. For convenience, these two interventions will from now on be referred to as suprathreshold PA_mono_-PAS and suprathreshold PA_bi_-PAS respectively. Sixteen MEPs were elicited every 5–7 s at an intensity of 120% RMT pre, immediately post, and 30 min post the intervention. The mean peak-to-peak amplitude was later extracted and used as an indication of excitability changes in the corticospinal projections to the TA.

### Experiment 1b: Assessment of Spinal Excitability

To investigate whether any changes occurred at the spinal level following the suprathreshold PA_bi_-PAS intervention, stretch reflexes were elicited prior to and following the intervention in nine participants (6 males, 3 females, age 26 ± 3 years). The right leg was affixed to a servo-controlled hydraulic actuator (MTS-systems Corporation, 215.35; Voigt et al., 1999), such that the anatomical ankle axis of rotation was closely aligned with the fulcrum of the actuator. The foot segment of the right leg of the participant was firmly strapped to a custom made plate that extended from the actuator, thus producing a tight interface between the arm of the motor and the foot of the participant, ensuring that the movement of the actuator was transmitted solely to the ankle joint. The angular position of the actuator was monitored by an angular displacement transducer (Transtek, DC ADT series 600). The participants were asked to maintain a 5% MVC in their right TA while the perturbations were applied. The EMG level was displayed on a computer screen placed in front of the subject. The instructions to the subjects at all times were to maintain the 5% MVC EMG level without interfering with the imposed plantarflexion perturbation.

Thirty stretches were randomly applied at intervals ranging from 5 s to 7 s (velocity: 100°s^−1^ to 200°s^−1^; amplitude: 4° to 6°; hold-time: 460 ms). The angular velocity and the amplitude of the imposed perturbations were adjusted for each subject so that the amplitude of the three response peaks observed in the TA EMG trace were approximately the same and also similar to the amplitude of the TA MEP prior to the intervention.

The latencies of the first and third response peaks (termed short-latency reflex (SLR) and long-latency reflex (LLR) or alternatively M1 and M3 in the literature) were extracted from the data both prior to and immediately following the intervention. The RMS value of a window extending 10 ms on either side of SLR and LLR (thus 20 ms for each window in total) was calculated and used as an indication of the size of each of these components of the TA stretch reflex. In this way it was ensured that neither the SLR nor the LLR responses were contaminated by the second response which is observed in some participants following an imposed perturbations as applied here.

### Experiment 2: The Effect of Subthreshold Compared to Suprathreshold Biphasic TMS on PAS Induced Changes

To address the second aim of whether PAS using a subthreshold biphasic TMS was as effective as PAS using a suprathreshold biphasic TMS pulse, 10 participants (7 males, 3 females, age 25 ± 3 years) received two interventions spaced at least 1 week apart. Suprathreshold PA_bi_-PAS was administered as for Experiment 1a. Subthreshold PA_bi_-PAS differed from suprathreshold PA_bi_-PAS in that the TMS pulse intensity used during the intervention was 95% of AMT. The order in which the two interventions were administered was randomized. As for Experiment 1a, the mean peak-to-peak amplitude was assessed both prior to and immediately following each intervention.

### Statistical Analysis

For Experiment 1a, a two-way within-subjects analysis of variance (ANOVA) was employed to determine the effect of the intervention (suprathreshold PA_bi_-PAS and suprathreshold PA_mono_-PAS) across time (pre, post, and 30 min post) on the TA MEP amplitude. In Experiment 1b, paired *t*-tests (2-tailed) were employed to evaluate the effect of the suprathreshold PAS interventions on the TA SLR and LLR. For Experiment 2, a two-way within-subjects ANOVA was employed to assess changes in TA MEP amplitude, with intervention (suprathreshold PA_bi_-PAS and subthreshold PA_bi_-PAS) and time (pre, post, and post 30 min) as the within-subjects factors. The significance level was set at *p* < 0.05. Bonferroni corrections were applied to multiple *post hoc* comparisons to determine the locus of the differences. The adjusted alpha level for *post hoc* comparisons following a significant main effect of time was therefore set at *p* < 0.0167. If not otherwise stated, all data are given as mean ± standard deviation.

## Results

### Somatosensory Evoked Potentials and the Interstimulus Interval during PAS

Across all participants, the afferent volley arrived at the somatosensory cortex at on average 46 ms (range: 41–50 ms) following the CPN stimulation. Based on our previous study (Mrachacz-Kersting et al., 2007), a central processing of 6 ms was added. Depending on the participant, the TMS was therefore triggered between 47 ms and 56 ms following the CPN stimulus during the PAS intervention.

### Experiment 1a: The Effect of Suprathreshold Monophasic Compared to Suprathreshold Biphasic TMS on PAS Induced Changes

**Figure 2:**
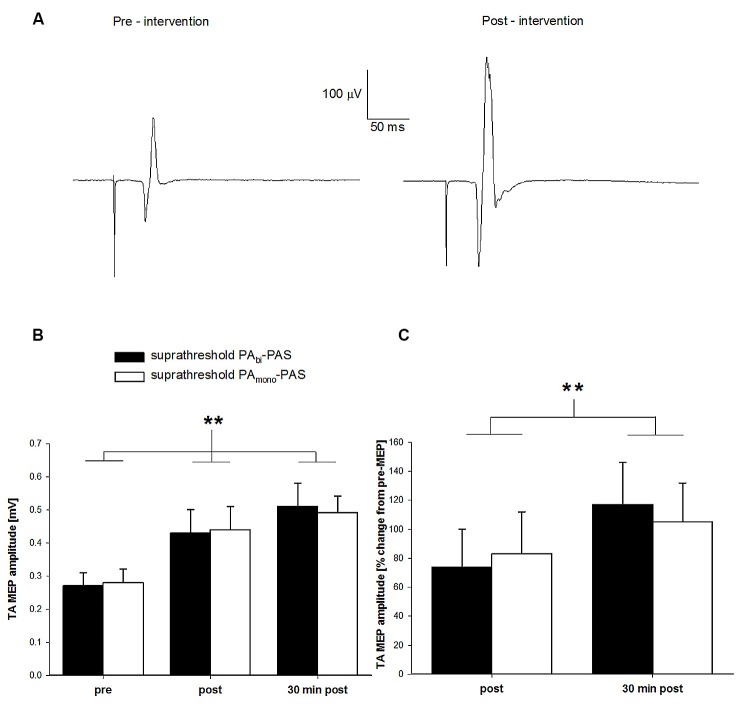
**Change in tibialis anterior (TA) motor evoked potential (MEP) amplitude after suprathreshold PA_bi_-paired associative stimulation (PAS) compared to suprathreshold PA_mono_-PAS at rest. (A)** TA MEP changes prior to and following the suprathreshold PA_bi_-PAS intervention for one participant. Data are the average of 16 trials. **(B)** TA MEP changes in mV, prior to, immediately following, and 30 min post suprathreshold PA_bi_-PAS (*n* = 11) compared to the suprathreshold PA_mono_-PAS (*n* = 11) intervention. Across both interventions, MEPs were significantly increased immediately after PAS (*p* = 0.018) and 30 min after PAS (*p* < 0.001) compared to before PAS. **(C)** TA MEP immediately following and 30 min post suprathreshold PA_bi_-PAS and suprathreshold PA_mono_-PAS interventions expressed as a percent change from values prior to the intervention. Asterisks in **(B,C)** denote significant differences. Error bars represent standard deviation.

In Figure 2A, the averaged (16 sweeps) raw TA MEP data prior to and after suprathreshold PA_bi_-PAS are presented for one participant. The raw MEP values were 0.24 mV and 0.51 mV, respectively. Across all participants, the mean pre-intervention TA MEP amplitude was 0.32 ± 0.23 mV, 0.43 ± 0.24 mV immediately post intervention, and 0.60 ± 0.33 mV 30 min after the intervention (Figure 2B). Immediately following the intervention, one participant failed to show an increase in the TA MEP amplitude, though 30 min post all participants exhibited an increase. For the suprathreshold PA_mono_–PAS, the TA MEP amplitudes were 0.28 ± 0.14 mV pre-intervention, 0.44 ± 0.22 mV immediately post intervention, and 0.49 ± 0.18 mV 30 min post intervention (Figure 2B).

The two-way within-subjects ANOVA revealed a significant main effect of time across both suprathreshold PAS interventions (*F*_(2,20)_ = 18.12, *p* < 0.001). *Post hoc* analyses revealed that the TA MEPs were significantly larger immediately following (0.44 ± 0.20 mV; *p* = 0.018) and 30 min after the interventions (0.50 ± 0.17 mV; *p* < 0.001) compared with pre-intervention values (0.27 ± 0.11 mV), and that the TA MEPs were not significantly different 30 min after the interventions compared with immediately post-intervention (*p* = 0.156). There was no significant main effect of intervention (*F*_(1,10)_ < 0.00, *p* = 0.99) nor a significant time by intervention interaction (*F*_(2,20)_ = 0.19, *p* = 0.83), indicating that both suprathreshold PAS interventions were effective at increasing the TA MEP amplitude.

Figure 2C contains the normalized TA MEP amplitude immediately following and 30 min after the cessation for both interventions expressed as a percent change from pre-intervention values (a value of zero indicates no change from pre) for all participants. On average, the TA MEP amplitude increased by 74% (range: 9%–250%) immediately following and by 117% (range: 4%–300%) 30 min following the cessation of the suprathreshold PA_bi_-PAS intervention and by 83% (range: 9%–335%) and 105% (range: 35%–265%) for the suprathreshold PA_mono_-PAS intervention.

### Experiment 1b: Assessment of Spinal Excitability

**Figure 3:**
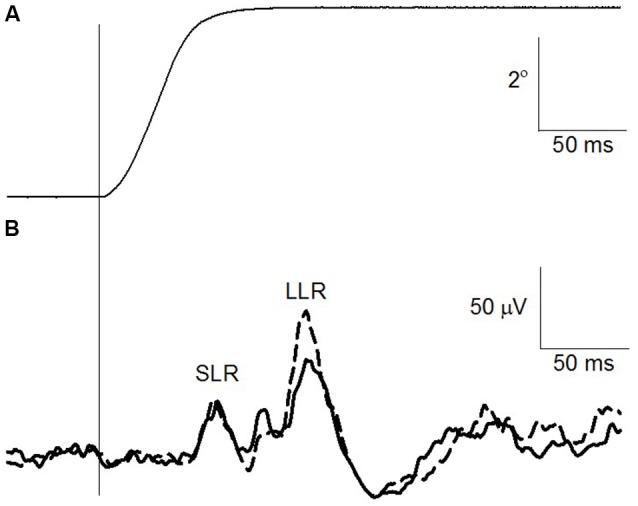
**Change in TA stretch reflex after suprathreshold PA_bi_-PAS at rest. (A)** Right ankle angle (°). The vertical line indicates the onset of the imposed plantarflexion perturbation. **(B)** TA rectified electromyographic (EMG) trace prior to (solid line) and following (dashed line) the suprathreshold PA_bi_-PAS intervention. Data are the average for 30 traces.

The change in ankle angle as well as the EMG recording of the TA both prior to and following the suprathreshold PA_bi_ PAS intervention is shown for one participant in Figures 3A,B, respectively. Each trace is the mean of 30 imposed rotations. The TA responds with three discernible peaks as seen in the EMG trace (Figure 3B). Care was taken to ensure that the amplitude of all three components was reasonably similar prior to the intervention and similar to the size of the TA MEP evoked in the pre-intervention measures.

Across all participants, the TA MEP amplitude increased following suprathreshold PA_bi_ PAS without a significant increase in the SLR (*t*_(8)_ = 0.93, *p* = 0.37) component of the TA stretch reflex. The LLR, which is at least in part of cortical origin (Petersen et al., 1998), increased on average by 117% across all participants, however this increase was not statistically significant (*t*_(8)_ = −0.66, *p* = 0.52). The background level of activation during the imposed plantar flexion perturbations did not differ significantly pre and post the intervention (*t*_(8)_ = 1.21, *p* = 0.25).

### Experiment 2: The Effect of Subthreshold Compared to Suprathreshold Biphasic TMS on PAS Induced Changes

**Figure 4:**
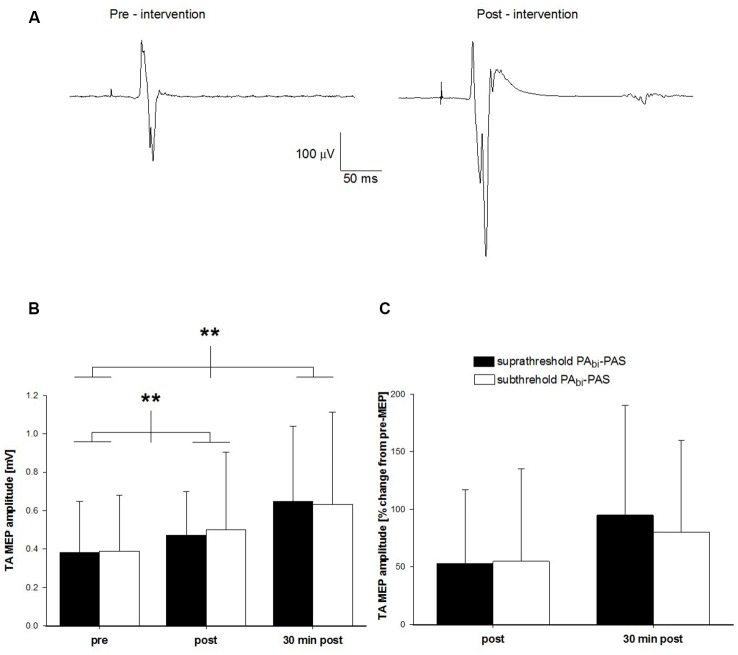
**Change in TA MEP amplitude after subthreshold PA_bi_-PAS compared to suprathreshold PA_bi_-PAS at rest. (A)** TA MEP changes prior to and following the subthreshold PA_bi_-PAS intervention for one participant. Data are the average of 16 trials. **(B)** TA MEP changes in mV, prior to, immediately following, and 30 min post suprathreshold PA_bi_-PAS compared to subthreshold PA_bi_-PAS intervention (*n* = 10). Across both interventions, MEPs were significantly increased immediately after PAS (*p* = 0.028) and 30 min after PAS (*p* = 0.002) compared to before PAS. **(C)** TA MEP immediately following, and 30 min post suprathreshold PA_bi_-PAS and subthreshold PA_bi_-PAS interventions expressed as a percent change from values prior to the intervention. Asterisks in **(B,C)** denote significant differences. Error bars represent standard deviation.

Across all participants, the RMT was 49 ± 8% S.O. and the AMT 41 ± 8% S.O. The averaged (16 sweeps) raw TA MEP data prior to and following subthreshold PA_bi_-PAS for one participant is displayed in Figure 4A. The raw TA MEP was 0.20 mV prior to and 0.65 mV following the intervention.

Across all participants, the pre-intervention mean TA MEP amplitude was 0.37 ± 0.26 mV for suprathreshold PA_bi_-PAS and 0.39 ± 0.29 mV for subthreshold PA_bi_-PAS. Immediately following the intervention, these values were 0.46 ± 0.22 mV and 0.50 ± 0.39 mV for suprathreshold PA_bi_-PAS and subthreshold PA_bi_-PAS, respectively, while 30 min post-intervention they were 0.62 ± 0.37 mV and 0.60 ± 0.47 mV (Figure 4B). On average, the TA MEP amplitude increased by 53% and 55% immediately following and by 95% and 80% 30 min following the cessation of the suprathreshold PA_bi_-PAS and subthreshold PA_bi_-PAS interventions, respectively, compared to the pre-intervention values (Figure 4C).

The two-way within-subjects ANOVA revealed that there was a significant main effect across the factor time (*F*_(2,18)_ = 14.21, *p* < 0.001). *Post hoc* analyses revealed that the TA MEPs were significantly larger immediately following (0.50 ± 0.08 mV; *p* = 0.028) and 30 min after the intervention (0.62 ± 0.11 mV; *p* = 0.002) compared with pre-intervention values (0.37 ± 0.08 mV). The TA MEPs were not significantly different immediately following compared with 30 min after the interventions (*p* = 0.13). There was no significant difference between the two interventions (*F*_(1,9)_ = 0.08, *p* = 0.79) or a significant interaction between intervention and time (*F*_(2,18)_ = 0.28, *p* = 0.76), indicating that both the suprathreshold PA_bi_-PAS and subthreshold PA_bi_-PAS interventions were effective at increasing the TA MEP amplitude.

Immediately following the intervention, two participants failed to show an increase in the TA MEP amplitude when suprathreshold PA_bi_-PAS was applied and one when subthreshold PA_bi_-PAS was applied. At 30 min post intervention, all participants exhibited an increase for suprathreshold PA_bi_-PAS while the MEP amplitude of the one participant remained unchanged following the subthreshold PA_bi_-PAS.

### Effects of Experimental Paradigms on the Antagonist SOL

As a stimulation applied to the area of the motor cortex associated with the TA also evoked a response in the SOL, we chose to monitor this muscle throughout the experimental sessions for possible changes. However, as in our previous study (Mrachacz-Kersting et al., 2007), no significant changes in the amplitude of the SOL MEP were found following any of the interventions (all *p*’s > 0.05).

## Discussion

In the past we have demonstrated that suprathreshold PA_mono_–PAS induces long lasting increases in the excitability of the cortical projections to the target muscle (Mrachacz-Kersting et al., 2007). The present study is the first in which PAS was applied targeting the TA, using a biphasic TMS stimulation pulse. The results show that suprathreshold PA_bi_-PAS can significantly increase the excitability of the cortical projections to the TA similar to suprathreshold PA_mono_-PAS. In addition, subthreshold PA_bi_-PAS applied with the TA at rest is able to induce similar changes in the TA MEP amplitude. In past experiments using upper limb muscles and a monophasic TMS pulse with an AP direction, this has only been possible in pre-activated muscles (Kujirai et al., 2006). This may have important consequences for PAS as a rehabilitative tool in patients unable to fully activate their TA such as occuring during foot drop following stroke.

### Experiment 1a: The Effect of Suprathreshold Monophasic Compared to Suprathreshold Biphasic TMS on PAS Induced Changes

The magnitude of change in the TA MEP amplitude following suprathreshold PA_bi_-PAS was on average 61% compared to pre-intervention and this effect was further enhanced 30 min after the cessation of the intervention to 123%. In contrast, the time course of the effects following PA_mono_-PAS were quantified as an 83% immediately following the intervention and 105% after 30 min had elapsed. In the past, changes of 96% and 88% immediately following and 30 min after the cessation of the intervention have been reported following suprathreshold PA_mono_-PAS (Mrachacz-Kersting et al., 2007). It thus appears that both suprathreshold PAS applied using a monophasic and a biphasic TMS pulse waveform are effective in increasing the excitability of the cortical projections to the TA.

One main difference between the changes following suprathreshold PA_bi_-PAS compared to suprathreshold PA_mono_-PAS is that in some participants (*n* = 3), the effect on the TA MEP was only visible 30 min following the cessation of the intervention. Similar differences in the time course for the effects of an intervention using either a biphasic or a monophasic TMS pulse form have been reported previously (Taylor and Loo, 2007). These authors used rTMS to induce a depression as quantified by a decrease in the size of the MEP in the FDI and reported that using a monophasic pulse form caused a depression immediately following the cessation of rTMS, though a depression was not seen until 20 min following the cessation of the intervention when a biphasic pulse was implemented. Results from rTMS interventions may not be directly related to those following PAS. However, as mentioned previously, a biphasic current will activate neurons within the cortex that are orientated in the AP as well as the PA direction. It may be speculated that this type of pulse form may not activate the same neural elements as a monophasic pulse form and indeed evidence on I wave recordings from the epidural space of humans confirms this (Di Lazzaro et al., 2001a). Thus, the complex waveform of the biphasic TMS may have repeatedly activated a different set or a different balance between excitatory and inhibitory interneurons compared to monophasic TMS as also suggested by Arai et al. (2007). If the excitatory neurons that are activated by a biphasic pulse (but not by a monophasic pulse) remain activated for a longer time than the corresponding inhibitory neurons, it may explain why the effect of PAS with a biphasic TMS can keep on developing, though this requires further investigation.

### Experiment 2: The Effect of Subthreshold Compared to Suprathreshold Biphasic TMS on PAS Induced Changes

Kujirai et al. (2006) and Roy et al. (2007) demonstrated that it was possible to facilitate the excitability of the cortical projections to FDI and TA, respectively, using PAS with subthreshold TMS. Methodological differences between the studies make direct comparisons difficult. In the present study, PAS was applied at rest with an ISI based on coincident synaptic input. Kujirai et al. (2006) applied PAS during a sustained voluntary contraction of the target muscle. It is not possible from that study to distinguish the effect of subthreshold stimulation from the effect of voluntary contraction in the MEP amplitude as voluntary contraction may enhance the effect of PAS (Mrachacz-Kersting et al., 2007). Roy et al. (2007) found that subthreshold PAS with a 20 ms 100 Hz train of CPN stimulation and arrival of the afferent inputs to the motor cortex after a TMS pulse facilitated the TA MEP amplitude. Repetitive electrical stimulation of the CPN alone has been shown in the past to facilitate the TA MEP, although with trains of pulses delivered at 0.33–1 Hz (Khaslavskaia et al., 2002; Knash et al., 2003; Khaslavskaia and Sinkjaer, 2005). In the present study, the single CPN electrical stimulus was delivered at motor threshold while Roy et al. (2007) used an intensity of 300% sensory perception threshold (which approximately equals motor threshold—own observation). The delivery of three electrical stimuli at the CPN around motor threshold may have a summation effect of the pulses such that the pulse train effectively corresponds to a suprathreshold stimulus. Nevertheless, similar to Kujirai et al. (2006), the current study provides evidence that when the TMS intensity as part of the PAS protocol is set below threshold for evoking a MEP in the target muscle, a significant increase in the excitability of the cortical projections to the TA occurs.

It was surprising that subthreshold PA_bi_-PAS induced similar increases in TA MEP amplitude compared to suprathreshold PA_bi_-PAS. It may be that the structures important in the induction of PAS have a low threshold to stimulation, though this is purely speculative and cannot be answered from data of the current study. However, Rotem and Moses (2008) investigated the effect of magnetic stimulation on one dimensional mammalian cell cultures. These authors were able to standardize the arrangements of the neurons and reported that aside from the orientation of the neurons, the neurons specific morphological and electrophysiological properties played a fundamental role in their activation through magnetic stimulation.

### Experiment 1b: Assessment of Spinal Excitability

The results from the stretch reflex data suggest that spinal excitability (assessed by the SLR of the stretch reflex) was unchanged, indicating a *supra* spinal origin of the TA MEP increase following PAS. However, recent reports have argued for changes at the spinal level when the human forearm muscle flexor carpi radialis (FCR) is targeted by PAS (Meunier et al., 2007). The authors suggest an altered pre-synaptic inhibition following PAS delivered to FCR concomitant to the alterations in H-reflex. Pre-synaptic inhibition may have a cortical origin and it is well known that the H-reflex and stretch reflex have different sensitivities to pre-synaptic inhibition (Morita et al., 1998).

The tendency for the LLR component of the TA stretch reflex to increase in amplitude following the intervention supports a cortical origin of the changes following PAS. Past studies indicate that the LLR component is at least in part of cortical origin (Petersen et al., 1998; Christensen et al., 2001; Wallace and Miles, 2001; van Doornik et al., 2004). Further, it is the LLR which is the dominant response in the TA when a sudden externally applied ankle dorsiflexion is induced during the stance phase of human walking (Christensen et al., 2001), suggesting it has a functional role in balance regulation during walking. Facilitation in the LLR component of the TA stretch reflex through a PAS protocol may thus have functional consequences across tasks, though this is speculative at this point and requires further investigation.

### Methodological Considerations

During the pre and post measures of MEP amplitude, a biphasic pulse type was used for those experiments that also used a biphasic TMS pulse shape during the intervention. Conversely, a monophasic pulse shape was used for those experiments that also used a monophasic TMS pulse shape during the intervention. The TA MEP amplitudes are thus not directly comparable. However, two previous studies investigating the effect of monophasic and biphasic low frequency rTMS on the amplitude of the FDI muscle MEP have found significant changes only when a monophasic pulse waveform was used during the intervention; one study used a monophasic pulse form for all pre and post measures (Taylor and Loo, 2007), while the other was similar to our study (Sommer et al., 2002). It is thus conceivable that if we had used the same pulse form for all of our baseline measures, similar results would have been attained.

## Conclusion

The aims of this study were to establish whether PAS applied using a biphasic pulse shape is able to induce changes in the excitability of the cortical projections to the TA and, whether it is possible to attain significant alterations in TA MEP amplitude when a subthreshold biphasic TMS pulse is used while the target muscle was at rest. This may have important implications for the rehabilitation of patients who have suffered a lesion of the corticospinal tract as it has been shown that the connectivity of this tract is associated with functional improvements (Thomas and Gorassini, 2005). TMS using a biphasic pulse shape will activate neurons and nerve bends orientated in various directions compared to a monophasic pulse shape. This may lead to the activation of a more diverse set of neurons. It is likely that neurons situated at multiple places in the motor cortex contribute to a particular movement and TMS with a biphasic shape using a double-cone coil may activate more of these locations. This may be useful when PAS is to be used in a rehabilitation setting.

## Author Contributions

NM-K and AJTS conceptualized and designed the study. NM-K collected the data partly with a student group, analyzed the data and drafted the manuscript, AJTS completed the statistical analysis, commented on the manuscript and approved the final version.

## Funding

This study was supported by the Spar Nord Foundation, and the Obelske Familiefond of Denmark.

## Conflict of Interest Statement

The authors declare that the research was conducted in the absence of any commercial or financial relationships that could be construed as a potential conflict of interest.
